# Effect of perioperative management on early graft function in living donor paediatric kidney transplantation

**DOI:** 10.1007/s00467-024-06520-4

**Published:** 2024-09-16

**Authors:** Jennifer Q. J. Zhang, Elena Cavazzoni, Anne M. Durkan, Deirdre Hahn, Hugh McCarthy, Stephen Alexander, Gordon Thomas, Sean E. Kennedy, Rachael Kermond, Justin Skowno, Ian Miles, Siah Kim

**Affiliations:** 1 Sydney Medical Program, The University of Sydney, Camperdown, Sydney, Australia; 2 Department of Nephrology, The Children’s Hospital at Westmead, Sydney, Australia; 3 Paediatric Intensive Care Unit, The Children’s Hospital at Westmead, Sydney, Australia; 4 School of Paediatrics and Child Health, The University of Sydney, Camperdown, Sydney, Australia; 5 Centre for Kidney Research, The Children’s Hospital at Westmead, Sydney, Australia; 6 Department of Surgery, The Children’s Hospital at Westmead, Sydney, Australia; 7 Department of Nephrology, Sydney Children’s Hospital Randwick, Sydney, Australia; 8 School of Clinical Medicine, UNSW Sydney, Sydney, Australia; 9 Department of Nephrology, Women’s and Children’s Hospital, North Adelaide, Adelaide, Australia; 10 Department of Anaesthesia, The Children’s Hospital at Westmead, Sydney, Australia; 11 School of Public Health, The University of Sydney, Camperdown, Sydney, Australia

**Keywords:** Paediatric kidney transplant, Intraoperative, Postoperative, Graft function, Fluid, Vasoactive agent

## Abstract

**Background:**

Paediatric kidney transplantation has an increased risk of surgical and vascular complications, with intensive care monitoring required postoperatively. This study aimed to determine if perioperative management affects early graft function in living donor paediatric kidney transplantation.

**Methods:**

Clinical data was extracted from the electronic medical record for living donor kidney transplants at two paediatric centres covering the state of New South Wales (NSW), Australia from 2009 to 2021. Estimated glomerular filtration rate (eGFR) of 7 days and 1-month post-transplant were calculated as measures of early graft function.

**Results:**

Thirty-nine eligible patients (female *n* (%) 13 (33%)) with a median (IQR) age of 6 (3–9) years and pre-transplant eGFR of 7 (6–10) mL/min/1.73 m^2^ were analysed. Mean (SD) central venous pressure (CVP) after revascularisation was 11 (4) mmHg. Intraoperatively, mean volume of fluid administered was 84 (39) mL/kg, and 34 (87%) patients received vasoactive agents. Average systolic blood pressure (BP) in the first 24-h post-transplant was 117 (12) mmHg. Postoperatively, median volume of fluid administered in the first 24 h was 224 (159–313) mL/kg, and 17 (44%) patients received vasoactive agents. Median eGFR 7 days and 1-month post-transplant were 115 (79–148) and 103 (83–115) mL/min/1.73 m^2^, respectively. Linear regression analyses demonstrated that after adjusting for age, the average CVP after revascularisation and average systolic BP in the first 24-h post-transplant were not associated with eGFR in the first month post-transplant.

**Conclusions:**

Targeted intraoperative and postoperative fluid and haemodynamic characteristics were achieved but did not correlate with early graft function.

**Graphical Abstract:**

**Figure:**
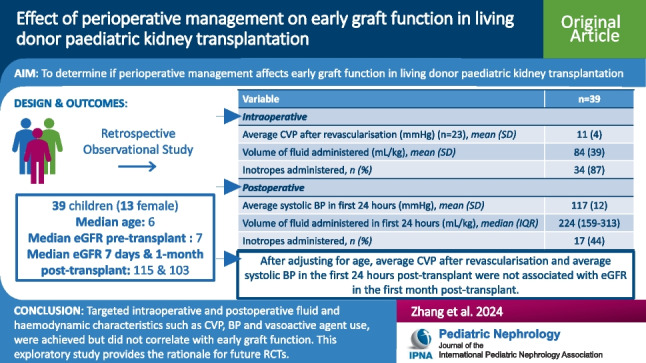
A higher resolution version of the Graphical abstract is available as Supplementary information.

**Supplementary Information:**

The online version contains supplementary material available at 10.1007/s00467-024-06520-4.

## Introduction

Kidney transplantation is the preferred treatment for children and adolescents with kidney failure [1]. Living organ donation is preferred due to higher graft survival rates of 98% and 94% at 1 and 5 years, respectively, compared to 93% and 77% for deceased donor grafts [2], and allows for avoidance or reduced time on dialysis and its associated morbidities [3]. However, there are challenges associated with paediatric kidney transplantation due to factors such as donor-recipient graft size mismatch, donor size and anatomy [1, 4]. As a result, kidney transplantation in the paediatric population requires expert invasive monitoring during and after the procedure [5]. Previous studies have highlighted how perioperative factors contribute to long-term graft survival [4, 5]. Rodricks et al. [5] reported that children who experienced early surgical complications had a higher incidence of graft failure. Inadequate perfusion to the graft leads to increased risk of delayed function [6], and therefore, interventions during the perioperative period are key contributors to positive transplant outcomes.

There is a lack of literature and evidence-based guidelines for perioperative management in paediatric kidney transplantation due to the smaller number of transplants performed and a subsequent lack of available data [7], with most protocols derived from adult clinical data [1]. Previous studies have highlighted a lack of agreement in the intraoperative and postoperative management of paediatric kidney transplantation, with considerable variation in monitoring procedures, haemodynamic goals, fluid replacement type and volume, vasoactive agent use and choices for analgesia [8, 9]. During the perioperative period, children are often treated with intravenous fluid and vasoactive agents to maintain mean arterial pressure (MAP; target, 70 mmHg) and central venous pressure (CVP; target, 8–12 cm H_2_O), and thus, ensure sufficient allograft perfusion [6, 10, 11]. However, the limited number of studies investigating fluid administration in paediatric kidney transplantation have been conflicting [11, 12]. In a single-centre study, Porn-Feldman et al. [11] reported that fluid overload (> 80 mL/kg) was associated with a greater improvement in kidney function after living donor kidney transplantation. In contrast, Wyatt et al. [12] reported that higher perioperative fluid administration and higher post-transplant urine output in a group of 102 children transplanted in five centres were associated with reduced kidney function at day 7. Wyatt et al. [12] also highlighted significant variability in post-transplant fluid administration, ranging from a total of 122–1194 mL/kg in the first 72 h in children between the ages of 2 and 19. The role of intraoperative vasoactive agent administration is uncertain with delayed graft function being reported in both living and deceased donor grafts [13]. Therefore, further studies are required to inform intraoperative and postoperative haemodynamic goals for adequate graft perfusion, and more specifically, the impact of haemodynamic characteristics, mainly fluid and vasoactive agent administration, requires further exploration.

The aim of this study was to determine if perioperative management affects early graft function in living donor paediatric kidney transplantation. We hypothesised that intraoperative and postoperative characteristics during living donor paediatric kidney transplantation, such as CVP, blood pressure (BP), fluid administration and vasoactive agent use, will impact estimated glomerular filtration rate (eGFR) in the first month post-transplant.

## Methods

### Study design and population

A retrospective observational study was performed for children under 18 years of age who received a kidney-only transplant from a living donor at either of the two children’s hospitals providing kidney transplantation across the state of New South Wales (NSW), Australia, from 2009 to 2021. We chose to restrict our study to living donor recipients to limit the confounding effect of cold ischaemic time and donor age among deceased donor kidney transplant recipients on early graft function. Ethics approval was obtained from the Sydney Children’s Hospital Network Human Research Ethics Committee (2019/ETH05919). For existing patients of The Children’s Hospital at Westmead or Sydney Children’s Hospital, Randwick, consent was obtained at clinic visits from both the parent/guardian and child if they were deemed to have sufficient maturity and capacity. For patients who had transitioned to adult services, an opt-out approach was used given the low-risk nature of the study. Patients were given 4 weeks to return an opt-out consent letter if they wished to be excluded.

### Transplant protocols

Each of the two participating centres had local transplant protocols which determined the perioperative perfusion BP, CVP and urine output targets and fluid replacement regimens. Generally, both centres target CVP of 8–12 perioperatively and a fluid replacement regimen of hourly ml for ml replacement of urine output in addition to insensible loss replacement [14, 15]. BP and urine output targets were individualised for each recipient.

### Exposures

Average CVP after revascularisation was determined as a marker of intraoperative graft perfusion. All CVP recorded measurements from immediately after revascularisation of the graft kidney until the end of the operation were averaged. CVP was measured through the insertion of a central venous line in either the right or left internal jugular vein. Average systolic BP in the first 24 h postoperatively was determined as a marker of postoperative graft perfusion. BP measurements were recorded hourly in the paediatric intensive care unit (PICU), and measurements from 24 h after the end of the operation were averaged. BP over the first 24 h in PICU was measured through a mixture of arterial line, automated BP and manual BP measurements.

### Outcome

eGFR 7 days and 1-month post-transplant were calculated using the revised Schwartz equation [16], as measures of early graft function defined in previous studies [11–13].

### Covariates

Routine clinical data was extracted from the electronic medical record including Cerner PowerChart® and databases specific to PICU. Both electronic and scanned handwritten clinical notes, operation reports, anaesthetic records, flowsheets, discharge summaries, pathology and imaging reports were reviewed. The following covariates were obtained from the medical records:

1. *Donor and recipient characteristics* including age at transplant, gender, donor relationship, ABO compatibility, height, weight, cause of kidney failure and kidney function prior to transplant. The graft kidney length-to-recipient height ratio was also calculated from the ultrasound performed immediately postoperatively due to the effect of donor-recipient size mismatch on eGFR [17].
2. *Intraoperative characteristics* including site of implantation, operation time, warm and cold ischaemic time, fluid administration (volume and type), vasoactive agent administration, CVP and BP. CVP measurements from immediately before and after revascularisation were also extracted. As BP measurements were taken every 5 min intraoperatively, the recorded values every 30 min were extracted to calculate average systolic and diastolic BP after revascularisation. Any unplanned return to theatre was also recorded.
3. *Postoperative characteristics* including length of stay in PICU, weight on discharge from PICU, BP, fluid administration (volume and type), vasoactive agent administration, urine output, intubation and oxygen therapy requirements and pathology results. At both sites, patients were admitted to PICU in the immediate postoperative period for close monitoring according to hospital procedures [14, 15]. Ventilation required was defined as patients who were still intubated on admission to PICU. Pathology results extracted for the first 24 h postoperatively included the highest serum chloride and lowest serum bicarbonate, as well as the highest lactate and lowest pH from blood gas tests, due to the common complication of metabolic acidosis post-transplant [18].
4. *Serum creatinine and tacrolimus levels for 1-month post-transplant.* If multiple measurements were performed on a single day, an average was taken.

### Statistical analyses

All normally distributed continuous data were reported as mean (standard deviation (SD)) and non-normally distributed data as median (interquartile range (IQR)). Significance was defined as *p* < 0.05. Univariate linear regression analyses were used to test for associations between perioperative characteristics and eGFR 7 days and 1-month post-transplant. Multivariate linear regression analyses investigating the effect of average CVP after revascularisation and average BP in the first 24 h postoperatively on eGFR 7 days and 1-month post-transplant were also performed, after adjusting for the confounding effect of recipient age at transplant. All statistical analyses were performed using SPSS Statistics® Version 28.0.0.0 (2021, IBM Corporation, USA).

## Results

### Baseline characteristics

**Fig. 1:**
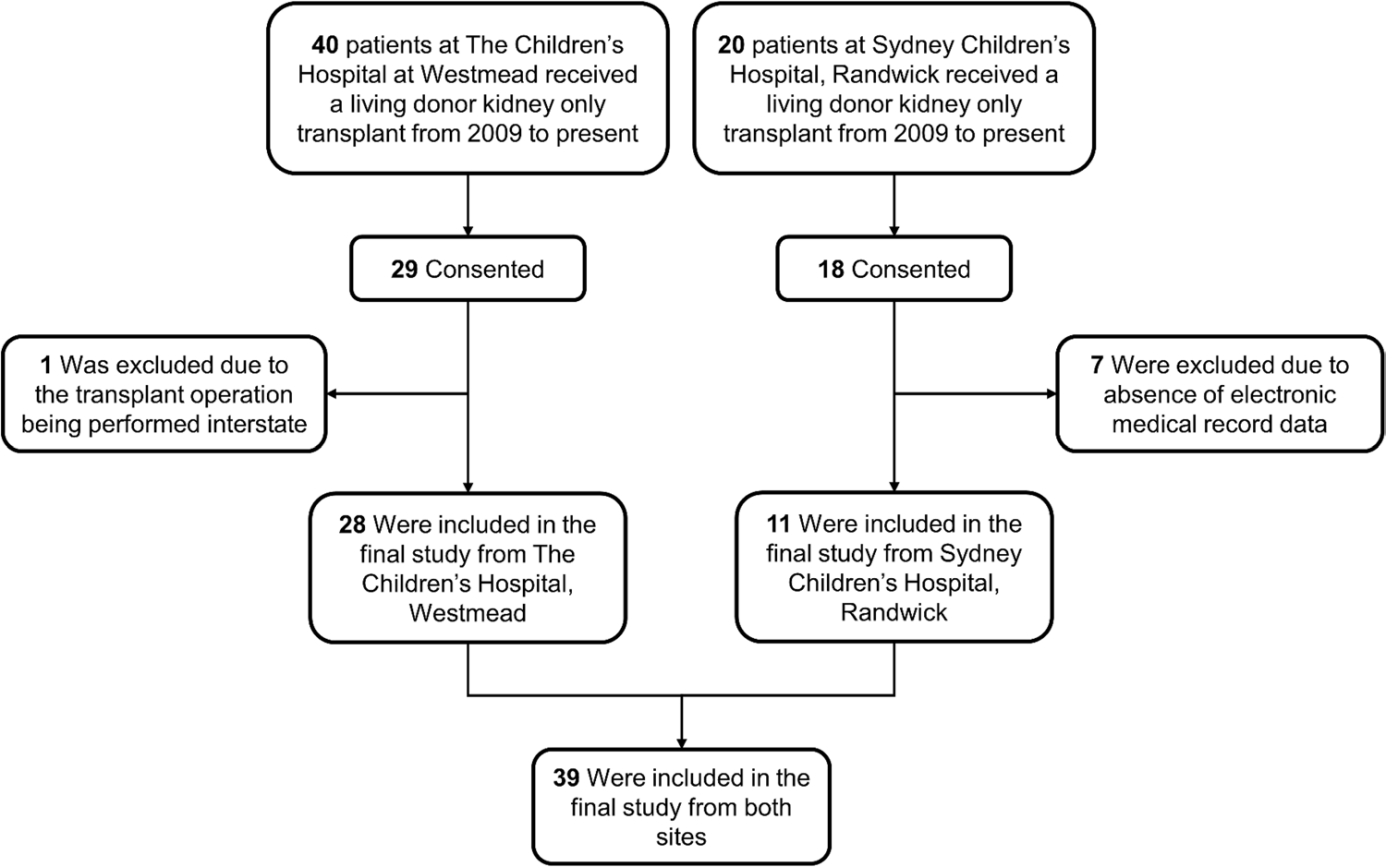
*Patient flow*. A total of 60 patients from The Children’s Hospital at Westmead and Sydney Children’s Hospital, Randwick, received living donor kidney-only transplants from 2009 to present. A total of 47 patients consented to participate in the study, and eight were excluded from the analysis. A total of 39 patients were included in the final analysis from both sites

As shown in Fig. 1, a total of 60 patients from The Children’s Hospital at Westmead and Sydney Children’s Hospital, Randwick, received living donor kidney-only transplants from 2009 to 2021. Forty-seven patients consented to participation in this study. Eight were excluded from the final analysis (Fig. 1), because the transplant operation was performed interstate (*n* = 1) and absence of electronic medical record data (*n* = 7).

A total of 39 patients were analysed, of whom 13 (33%) were female. The baseline clinical characteristics of the donors and recipients are described in Table 1. The mean (SD) donor age at transplant was 40 (8) years, and the most common donor relationship (*n*, %) was “Mother” (17, 44%), followed by “Father” (16, 41%). ABO-incompatible transplants were performed in 4 (10%). The median (IQR) recipient age at transplant was 6 (3–9) years, and the most common cause of kidney failure was congenital anomalies of the kidney and urinary tract (CAKUT) (14, 36%), followed by glomerular disease (13, 33%). Median weight prior to transplant was 18 (12–26) kg. Median eGFR prior to transplant was 7 (6–10) mL/min/1.73 m^2^. The mean graft kidney length-to-height ratio was 11 (2) %.

**Table 1:** Baseline characteristics of the donors and recipients of living donor paediatric kidney transplants at The Children’s Hospital at Westmead and Sydney Children’s Hospital, Randwick, from 2009 to present (*n* = 39). Data were collected from the electronic medical record

	Variables	*n* = 39
**Donor characteristics**	Age at transplant (years), *mean (SD)*	40 (8)
	Donor relationship, *n* (%) - Mother - Father - Other	17 (44) 16 (41) 6 (15)
	ABO Compatible, *n* (%)	35 (90)
**Recipient characteristics**	Female, *n* (%)	13 (33)
	Age at transplant (years), median (IQR)	6 (3–9)
	Height (cm), median (IQR)	108 (89–127)
	Weight (kg), median (IQR)	18 (12–26)
	Cause of kidney failure, *n* (%) - CAKUT - Glomerular disease - Cystic kidney disease - Other	14 (36) 13 (33) 6 (15) 6 (15)
	eGFR prior to transplant (mL/min/1.73 m^2^), median IQR	7 (6–10)
	Graft kidney length (cm), mean (SD)	11 (1)
	Graft kidney length: height (%), mean (SD)	11 (2)

*SD*, standard deviation; *IQR*, interquartile range; *CAKUT*, congenital anomalies of kidney and urinary tract; *eGFR*, estimated glomerular filtration rate

### Intraoperative characteristics

The data relating to intraoperative management in paediatric living donor kidney transplants is shown in Table 2. Most donor kidneys were implanted in the right extraperitoneal position (20, 51%), with 44% (17) implanted intraperitoneally. Mean operative time was 283 (66) min. Intraoperative CVP measurements were only available for patients at one hospital only (*n* = 23). Mean CVP remained consistent immediately before and after revascularisation, recorded as 11 (5) mmHg. In addition, average CVP after revascularisation until the end of the operation was 11 (4) mmHg. Similarly, intraoperative BP measurements were only available for a subset of 25 patients. Average systolic BP and diastolic BP after revascularisation until the end of the operation were 98 (13) mmHg and 49 (7) mmHg, respectively. Mean volume of fluid administered intraoperatively was 84 (39) mL/kg, with 82% (*n* = 32) receiving both crystalloid and colloid fluid type and 16 (41%) receiving blood products. The most common crystalloid fluid type was 0.9% sodium chloride, and the most common colloid fluid type was 4% serum albumin. Out of 34 patients who received vasoactive agents intraoperatively, most patients received metaraminol only (*n* = 24). The remaining patients received either dopamine only (*n* = 4), metaraminol and dopamine (*n* = 3), metaraminol and noradrenaline (*n* = 1), metaraminol and dobutamine (*n* = 1) or a combination of metaraminol, dobutamine and noradrenaline (*n* = 1).

**Table 2:** Intraoperative characteristics for living donor paediatric kidney transplants at The Children’s Hospital at Westmead and Sydney Children’s Hospital, Randwick, from 2009 to present (*n* = 39). Data were collected from the electronic medical record

Variables	*n* = 39
Site of implantation, *n* (%) - Right extraperitoneal - Right intraperitoneal - Left extraperitoneal - Left intraperitoneal	20 (51) 16 (41) 2 (5) 1 (3)
Operation time (min), mean (SD)	283 (66)
Cold ischaemic time (min) (*n*** = 10), mean (SD)**	162 (111)
Warm ischaemic time (min)(*n* = 35), median (IQR)	41 (35–46)
CVP immediately before revascularisation (mmHg) (*n* = 23), mean (SD)	11 (5)
CVP immediately after revascularisation (mmHg) (*n* = 24), mean (SD)	11 (5)
Average CVP after revascularisation (mmHg) (*n* = 23), mean (SD)	11 (4)
Average systolic BP after revascularisation (mmHg) (*n* = 25), mean (SD)	98 (13)
Average diastolic BP after revascularisation (mmHg)** (*n* = 25), mean (SD)**	49 (7)
Volume of fluid administered (mL/kg), mean (SD)	84 (39)
Fluid type, *n* (%) - Crystalloid and colloid - Crystalloid only - Colloid only	32 (82) 6 (15) 1 (3)
Blood products administered, *n*	16 (41)
Urine output intraoperatively (*n* = 31), *n*	30 (77)
Inotropes administered, *n* (%)	34 (87)
Unplanned return to theatre, *n*	6 (15)

*SD*, standard deviation; *IQR*, interquartile range; *CVP*, central venous pressure; *BP*, blood pressure

### Postoperative characteristics

The clinical and pathology data collected during the transplant recipients’ stay in PICU is shown in Table 3. Median length of stay in PICU was 2 (1–4) days, with longer length of PICU stay correlating with younger age at transplant (*p* < 0.01). The median increase in weight of patients on discharge from PICU compared to weight pre-transplant was 1.9 (1.5–3.9) kg, equating to a medium percentage weight gain of 11 (7–15) %. The average systolic BP in the first 24-h post-transplant was 117 (12) mmHg. The median volume of fluid administered in the first 24 h was 224 (159–313) mL/kg, with most receiving crystalloid fluid only (*n* = 38, 97%). The most common fluid type used was 0.9% sodium chloride. Median urine output in the first 24 h was 145 (91–230) mL/kg. Only three (8%) patients received blood products in PICU. Sixteen patients (41%) were still intubated on admission to PICU, with increasing duration of intubation in the PICU correlated with younger age (*p* = 0.02), and most patients required oxygen therapy during their stay (*n* = 28, 72%). Out of 17 patients who received vasoactive agents in PICU, 11 (28%) patients received vasoactive agents after the first hour. Most patients received dopamine only (*n* = 9). The remaining patients received either noradrenaline only (*n* = 3), metaraminol only (*n* = 1), metaraminol and noradrenaline (*n* = 1), dopamine and noradrenaline (*n* = 1) or the type of vasoactive agents administered was not recorded (*n* = 2). The mean highest recorded serum chloride level in the first 24 h was 112 (5) mmol/L. The mean tacrolimus levels over 7 days and 1-month post-transplant were 8.0 (2.5) and 8.7 (SD 1.4) µg/L, respectively. The tacrolimus data for one patient was excluded due to erroneous levels (> 50 µg/L). Two patients were reported to have pulmonary oedema, and three patients were reported to have clinically significant fluid overload during their hospital admission.

**Table 3:** Postoperative characteristics for living donor paediatric kidney transplants at The Children’s Hospital at Westmead and Sydney Children’s Hospital, Randwick, from 2009 to present (*n* = 39). Data was collected from the electronic medical record

	Variables	*n* = 39
**Postoperative characteristics**	Length of stay in PICU (days), median (IQR)	2 (1–4)
	Weight on discharge from PICU (kg) (*n* = 34), median (IQR)	20 (13–31)
	Percent increase in weight on discharge from PICU (%) (*n* = 34), median (IQR)	11 (7–15)
	Inotropes administered, *n* (%) - Inotropes administered in first hour only - Inotropes administered after first hour	17 (44) 6 (15) 11 (28)
	Intubation required, *n* (%)	16 (41)
	Epidural administered, *n* (%)	27 (69)
	Oxygen therapy required, *n* (%)	28 (72)
	Systolic BP on admission (mmHg), mean (SD)	120 (15)
	Diastolic BP on admission (mmHg), mean (SD)	70 (13)
	Average systolic BP in first 24 h (mmHg), mean (SD)	117 (12)
	Average diastolic BP in first 24 h (mmHg), median (IQR)	66 (61–69)
	Volume of fluid administered in first 24 h (mL/kg), median (IQR)	224 (159–313)
	Fluid type, *n* (%) - Crystalloid and colloid - Crystalloid only - Colloid only	1 (3) 38 (97) 0 (0)
	Blood products administered, *n* (%)	3 (8)
	Urine output in first 24 h (mL/kg), median (IQR)	145 (91–230)
**Pathology results**	Serum chloride – highest in first 24 h (mmol/L), mean (SD)	112 (5)
	Whole blood lactate – highest in first 24 h (mmol/L), median (IQR)	1.5 (1.1–2.1)
	pH – lowest in first 24 h, mean (SD)	7.28 (0.08)
	Serum bicarbonate – lowest in first 24 h (mmol/L), mean (SD)	17 (3)
	Average tacrolimus level over 7 days post-transplant (µg/L) (*n* = 38), mean (SD)	8.0 (2.5)
	Average tacrolimus level over 1-month post-transplant (µg/L) (*n* = 38), mean (SD)	8.7 (1.4)

*SD*, standard deviation; *IQR*, interquartile range; *PICU*, paediatric intensive care unit; *BP*, blood pressure

### Correlation between perioperative characteristics and early graft function

**Fig. 2:**
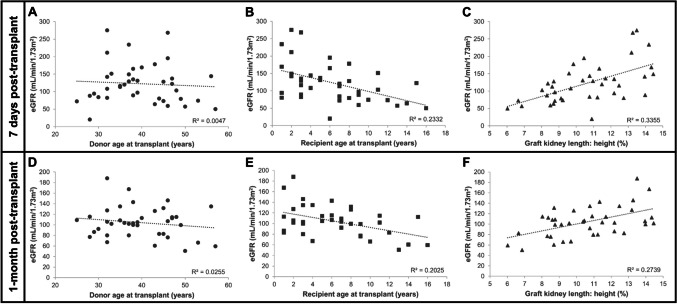
Scatter plots demonstrating the relationship between donor and recipient characteristics and estimated glomerular filtration rate (eGFR) 7 days and 1-month post-living donor paediatric kidney transplant (*n* = 39). Correlation between **A** donor age at transplant, **B** recipient age at transplant, **C** graft kidney length-to-height ratio and eGFR 7 days post-transplant. Correlation between **D** donor age at transplant, **E** recipient age at transplant, **F** graft kidney length-to-height ratio and eGFR 1-month post-transplant

Median eGFR 7 days and 1-month post-transplant were 115 (79–148) and 103 (83–115) mL/min/1.73 m^2^, respectively. The relationship between continuous baseline, intraoperative and postoperative characteristics, and eGFR after 7 days and 1-month post-transplant are shown in Fig. 2 and Supplemental Fig. S1 and S2, respectively.

#### Univariate linear regression analyses

At 7-day post-transplant, univariate linear regression analyses demonstrated that increasing recipient age at transplant was associated with lower eGFR (1-year increase in age, − 6.60 mL/min/1.73 m^2^, 95% CI (− 10.59, − 2.60), *p* < 0.01) and increasing graft kidney length-to-recipient height ratio was associated with higher eGFR (one % increase, 14.43 mL/min/1.73 m^2^, 95% CI (7.65, 21.21), *p* < 0.01) (Table 4; SI Fig. 2B–C). These results were consistent at 1-month post-transplant (Table 5; SI Fig. 2E–F).

**Table 4:** Univariate linear regression analyses for donor, recipient, intraoperative and postoperative characteristics of living donor paediatric kidney transplants with estimated glomerular filtration rate (eGFR) 7 days post-transplant as the dependent variable (*n* = 39)

		*B*	95% CI	*p*
**Donor characteristics**	Age at transplant (years)	− 0.49	(− 2.85, 1.88)	0.68
	Donor relationship - Mother - Father - Other	− 45.32 − 17.06 −	(− 95.29, 4.64) (− 67.44, 33.31) −	0.13 - - -
**Recipient characteristics**	Gender	2.73	(− 37.12, 42.58)	0.89
	Age at transplant (years)	− 6.60	(− 10.59, − 2.60)	0.002*
	Cause of kidney failure - CAKUT - Glomerular disease - Cystic kidney disease - Other	− 16.00 − 2.54 21.67 −	(− 68.61, 36.61) (− 55.75, 50.67) (− 40.58, 83.91) −	0.57 - - - -
	Graft kidney length: height (%)	14.43	(7.65, 21.21)	< 0.001*
**Intraoperative characteristics**	Cold ischaemic time (min) (*n* = 10)	− 0.06	(− 0.51, 0.40)	0.79
	Warm ischaemic time (min) (*n* = 35)	− 0.24	(− 2.06, 1.59)	0.80
	CVP immediately after revascularisation (mmHg) (*n* = 24)	2.47	(− 2.46, 7.39)	0.31
	Average CVP after revascularisation (mmHg) (*n* = 23)	4.99	(− 0.74, 10.72)	0.09
	Average systolic BP after revascularisation (mmHg) (*n* = 25)	− 1.27	(− 2.84, 0.29)	0.11
	Average diastolic BP after revascularisation (mmHg) (*n* = 25)	− 1.62	(− 4.52, 1.27)	0.26
	Volume of fluid administered (mL/kg)	0.30	(− 0.18, 0.77)	0.21
	Blood products administered	22.05	(− 15.43, 59.54)	0.24
	Inotropes administered	28.61	(− 26.77, 84.00)	0.30
**Postoperative characteristics**	Length of stay in PICU (days)	19.95	(10.63, 29.28)	< 0.001*
	Percent increase in weight on discharge from PICU (%) (*n* = 34)	− 1.55	(− 3.72, 0.63)	0.16
	Inotropes administered	22.56	(− 14.58, 59.70)	0.23
	Intubation required	61.58	(29.36, 93.81)	< 0.001*
	Epidural administered	− 21.32	(− 61.40, 18.77)	0.29
	Average systolic BP in first 24 h (mmHg)	− 0.52	(− 2.12, 1.07)	0.51
	Average diastolic BP in first 24 h (mmHg)	− 0.77	(− 2.62, 1.08)	0.40
	Volume of fluid administered in first 24 h (mL/kg)	0.08	(− 0.08, 0.24)	0.33
	Blood products administered	− 28.61	(− 98.47, 41.25)	0.41
	Urine output in first 24 h (mL/kg)	0.04	(− 0.13, 0.21)	0.61
	Serum chloride – highest in first 24 h (mmol/L)	− 1.77	(− 5.64, 2.09)	0.36
	Average tacrolimus level over 7 days post-transplant (µg/L) (*n* = 38)	5.38	(− 1.71, 12.47)	0.13

*B*, unstandardised coefficient; *CI*, confidence intervals; *CAKUT*, congenital anomalies of kidney and urinary tract; *CVP*, central venous pressure; *BP*, blood pressure; *PICU*, paediatric intensive care unit

The asterisk symbol (*) indicates statistical significance *p* < 0.05

**Table 5:** Univariate linear regression analyses for donor, recipient, intraoperative and postoperative characteristics of living donor paediatric kidney transplants with estimated glomerular filtration rate (eGFR) 1-month post-transplant as the dependent variable (*n* = 39)

		*B*	95% CI	*p*
**Donor characteristics**	Age at transplant (years)	− 0.58	(− 1.77, 0.61)	0.33
	Donor relationship - Mother - Father - Other	− 25.30 − 17.91 −	(− 50.82, 0.23) (− 43.65, 7.83) −	0.15 - - -
**Recipient characteristics**	Gender	− 7.21	(− 27.37, 12.95)	0.47
	Age at transplant (years)	− 3.14	(− 5.21, − 1.06)	0.004*
	Cause of kidney failure - CAKUT - Glomerular disease - Cystic kidney disease - Other	− 15.96 − 10.42 − 4.13 −	(− 42.88, 10.95) (− 37.64, 16.80) (− 35.98, 27.71) −	0.65 - - - -
	Graft kidney length: height (%)	6.65	(3.04, 10.26)	< 0.001*
**Intraoperative characteristics**	Cold ischaemic time (min) (*n* = 10)	− 0.04	(− 0.25, 0.17)	0.67
	Warm ischaemic time (min) (*n* = 35)	− 0.27	(− 1.15, 0.62)	0.55
	CVP immediately after revascularisation (mmHg) (*n* = 24)	− 1.16	(− 3.33, 1.01)	0.28
	Average CVP after revascularisation (mmHg) (*n* = 23)	− 0.58	(− 3.32, 2.16)	0.67
	Average systolic BP after revascularisation (mmHg) (*n* = 25)	− 0.23	(− 0.99, 0.54)	0.55
	Average diastolic BP after revascularisation (mmHg) (*n* = 25)	− 0.44	(− 1.81, 0.93)	0.52
	Volume of fluid administered (mL/kg)	0.14	(− 0.10, 0.39)	0.24
	Blood products administered	8.73	(− 10.51, 27.96)	0.36
	Inotropes administered	4.46	(− 24.12, 33.05)	0.75
**Postoperative characteristics**	Length of stay in PICU (days)	7.39	(2.01, 12.68)	0.01*
	Percent increase in weight on discharge from PICU (%) (*n* = 34)	− 0.56	(− 1.66, 0.54)	0.31
	Inotropes administered	11.47	(− 7.44, 30.39)	0.23
	Intubation required	17.59	(− 0.97, 36.14)	0.06
	Epidural administered	− 5.73	(− 26.37, 14.92)	0.58
	Average systolic BP in first 24 h (mmHg)	− 0.02	(− 0.84, 0.79)	0.96
	Average diastolic BP in first 24 h (mmHg)	0.22	(− 0.73, 1.16)	0.65
	Volume of fluid administered in first 24 h (mL/kg)	0.04	(− 0.04, 0.12)	0.36
	Blood products administered	− 28.21	(− 62.87, 6.45)	0.11
	Urine output in first 24 h (mL/kg)	0.01	(− 0.08, 0.10)	0.80
	Serum chloride – highest in first 24 h (mmol/L)	− 0.40	(− 2.40, 1.60)	0.69
	Average tacrolimus level over 1-month post-transplant (µg/L) (*n* = 38)	− 0.13	(− 6.59, 6.32)	0.97

*B*, unstandardised coefficient; *CI*, confidence intervals; *CAKUT*, congenital anomalies of kidney and urinary tract; *CVP*, central venous pressure; *BP*, blood pressure; *PICU*, paediatric intensive care unit

The asterisk symbol (*) indicates statistical significance *p* < 0.05

Intraoperatively, an increase of one mmHg average CVP after revascularisation was associated with a 4.99 mL/min/1.73 m^2^ increase in eGFR on day 7 post-transplant, but this effect was not statistically significant (95% CI (− 0.74, 10.72), *p* = 0.09) (Table 4; SI Fig. S1A). There were no other associations between any intraoperative characteristics and eGFR 7 days and 1-month post-transplant (Tables 4 and 5; SI Fig. S1).

Postoperatively, increased length of stay in PICU correlated with higher eGFR both 7 days (1-day increase, 19.95 mL/min/1.73 m^2^, 95% CI (10.63, 29.28), *p* < 0.01) (Table 4; SI Fig. S2A) and 1-month post-transplant (1-day increase, 7.39 mL/min/1.73 m^2^, 95% CI (2.01, 12.68), *p* = 0.01) (Table 5; SI Fig. S2E) reflecting the association between age and length of stay in PICU. In addition, intubation still required postoperatively on admission to PICU was associated with higher eGFR 7 days post-transplant (Table 4), but this association was not significant at 1-month post-transplant (Table 5). Average tacrolimus level did not correlate with eGFR 7 days (1 µg/L increase, 5.38 mL/min/1.73 m^2^, 95% CI (− 1.71, 12.47), *p* = 0.13) (Table 4; SI Fig. S2D) or 1-month (1 µg/L increase, − 0.13 mL/min/1.73 m^2^, 95% CI (− 6.59, 6.32), *p* = 0.97) (Table 5; SI Fig. S2H) post-transplant.

There were no significant associations between BP intra- and postoperatively and eGFR post-transplant (Tables 4 and 5; SI Fig. S1B & E; Fig. S2B & F). In addition, volume of fluid administered and vasoactive agents use intra- and postoperatively were not associated with eGFR post-transplant (Tables 4 and 5; SI Fig. S1C & F; Fig. S2C & G).

#### Multivariable linear regression analyses

As recipient age at transplant was a potential confounder, multivariate linear regression analyses adjusted for age were performed (Table 6). In this analysis, average CVP after revascularisation, as a measure of intraoperative interventions, was not associated with eGFR 7 days (1 mmHg increase, 2.71 mL/min/1.73 m^2^, 95% CI (− 3.33, 8.75), *p* = 0.36) or 1-month (1 mmHg increase, − 1.80 mL/min/1.73 m^2^, 95% CI (− 4.63, 1.03), *p* = 0.20) post-transplant (Table 6). Similarly, average systolic BP in the first 24-h post-transplant, as a measure of postoperative interventions, did not correlate with eGFR 7 days (1 mmHg increase, 0.37 mL/min/1.73 m^2^, 95% CI (− 1.15, 1.90), *p* = 0.62) or 1-month (1 mmHg increase, 0.44 mL/min/1.73 m^2^, 95% CI (− 0.34, 1.22), *p* = 0.26) post-transplant (Table 6).

**Table 6:** Multivariate linear regression analyses for intraoperative and postoperative characteristics of living donor paediatric kidney transplants with estimated glomerular filtration rate (eGFR) 7 days and 1-month post-transplant as the dependent variables, adjusted for recipient age at transplant (*n* = 39)

		eGFR 7 days post-transplant			eGFR 1-month post-transplant		
		*B*	95% CI	*p*	*B*	95% CI	*p*
**Intraoperative characteristic**	Average CVP after revascularisation (mmHg) (*n* = 23)	2.71	(− 3.33, 8.75)	0.36	− 1.80	(− 4.63, 1.03)	0.20
**Postoperative characteristic**	Average systolic BP in first 24 h (mmHg)	0.37	(− 1.15, 1.90)	0.62	0.44	(− 0.34, 1.22)	0.26

*B*, unstandardised coefficient; *CI*, confidence intervals; *eGFR*, estimated glomerular filtration rate; *CVP*, central venous pressure; *BP*, blood pressure

## Discussion and conclusions

There is still wide variation in the intraoperative and postoperative management of paediatric kidney transplantation, with a paucity of evidence-based guidelines on fluid management or vasoactive agents use [8, 9]. The main purpose of this study was to determine the effect of perioperative management on early graft function in living donor paediatric kidney transplantation. This study demonstrated that after adjusting for age, average CVP after revascularisation and average systolic BP in the first 24-h post-transplant, as markers of intraoperative and postoperative care, respectively, were not associated with early graft function. Recipient age at transplant, graft kidney length-to-recipient height ratio, length of stay in PICU and intubation still required in PICU were associated with early graft function. However, no correlations between specific intraoperative and postoperative interventions such as volume of fluid administered, vasoactive agents use and early graft function were identified. It is, however, important to note that strict targets for CVP and BP were maintained according to protocols during the perioperative period [14, 15], as highlighted by minimal variability in these parameters in our study. Therefore, as no negative associations with early graft function were demonstrated, these data suggest that current practices to maintain haemodynamic goals intra- and postoperatively are likely achieving appropriate outcomes for living donor paediatric kidney transplantation.

The most significant predictors of early graft function in this study population were recipient age at transplant and graft kidney length-to-height ratio. The negative association between recipient age and early graft function is most likely a confounding effect of using the revised Schwartz equation to calculate eGFR [16]. This formula is known to potentially overestimate kidney function in lower body weights and underestimate at higher body weights [19, 20]. It is also well known that adult donor organs require a significant amount of circulating blood volume when transplanted [13]. Therefore, the positive association between graft kidney length-to-height ratio is likely due to the graft kidney hyperfiltrating in the early postoperative period due to its comparatively larger size [13].

The importance of haemodynamic monitoring focused on BP and fluid status during the perioperative period for optimal graft perfusion has been highlighted previously [6, 10, 21]. In adult populations, conflicting findings have been reported on the relationship between intraoperative CVP and graft function [21–24]. Previous studies have reported CVP as an unreliable marker of fluid status [6], as it is influenced by multiple other factors including pericardial, intrathoracic and intra-abdominal pressure and arterial and venous sufficiency [25], which may explain the lack of correlation between intraoperative CVP and early graft function in our study. This finding may also be related to the fact that a narrow range of 8–12 mmHg is targeted for CVP intraoperatively [14, 15]. In contrast, Michelet et al. [24] demonstrated a strong positive correlation between mean BP-to-weight ratio 10 min after reperfusion and postoperative creatinine clearance at day 1 in a cohort of 102 paediatric kidney transplant patients. However, our study showed no correlation between average intraoperative systolic and diastolic BP after revascularisation with early graft function.

Previous studies in adults have associated hypovolaemia with delayed graft function [11], and thus, adequate fluid status in the perioperative period is vital [6]. However, the high CVP and “supranormal” circulation targeted in paediatric patients leads to an increased risk of pulmonary oedema and requiring ventilation postoperatively [9, 13, 24]. In addition, excessive fluid administration can lead to hypertension in the early postoperative period [26], and is associated with increased risk of acute kidney injury and mortality [27]. The mean volume of fluid administered intraoperatively in our study was 84 mL/kg, which is comparable to other paediatric studies [12, 28]. However, the median volume of fluid administered postoperatively in the first 24 h for our study was 224 mL/kg. Although this is much higher than in other paediatric studies this is likely explained by our restriction to living donor kidney transplants as the volume of fluid administered is higher than for deceased donor transplants, reflecting higher urine production [29]. Previous studies regarding perioperative fluid volume management in paediatric kidney transplants remain conflicting [11, 12]. This study found no association between volume of fluid administered intra- or postoperatively with early graft function. However, there was a median percent increase in weight of 11% on discharge from PICU compared to pre-transplant reported in this study population. In addition, a considerable proportion of patients (72%) required oxygen therapy during their PICU admission. Together, these data suggest more patients in this study may have been fluid-overloaded than reported in their discharge summaries [30, 31]. Though aggressive fluid management is crucial to reduce the risk of kidney graft thrombosis [32], the volume of fluid administered in the postoperative period may need to be better individualised, given fluid overload defined as > 10% weight gain is associated with increased mortality and adverse outcomes [31].

Limited studies regarding the effect of postoperative care on early graft function in paediatric kidney transplantation have been conducted [6]. This study found that postoperatively, increased length of stay in PICU, and intubation still required on admission to PICU, were associated with higher eGFR post-transplant. This finding is most likely related to the confounding effect of recipient age at transplant, where younger and smaller patients are more likely to have higher eGFR, prolonged stays, and require intubation postoperatively [6] and also likely reflect that smaller children are more prone to temporary overhydration which contributes to a longer period of ventilation. However, this finding may also suggest that more frequent monitoring of CVP, BP and fluid status provided by PICU in the early postoperative period may improve early graft outcomes. The lack of correlation between other postoperative characteristics and early graft function in our study suggests that aggressive intervention, particularly related to fluid and vasoactive agents administration, in the postoperative period may not be necessary for adequate graft perfusion, however, further exploration is required.

A strength of this study was its focus on patients who received a living donor transplant, as most previous studies in perioperative management have analysed living and deceased donor cohorts together [13, 23, 24]. These results are often confounded by the effect of donor characteristics and cold ischaemic time on early graft function in deceased donor transplants [11, 23, 24]. With living donors being preferred in a paediatric setting [3], it is important to investigate the effect of the perioperative period in this population specifically. On the other hand, it is possible that because deceased donor grafts have higher risks of delayed graft function, optimal perioperative care could be found to be even more important to short- and long-term outcomes. Another strength is the inclusion of patients from the two major paediatric centres in NSW, Australia, increasing the applicability of the study findings. The main limitation of this study was the small sample size of 39 patients, where a larger sample would be required for robust and generalisable conclusions. However, it is important to note that a limited number of paediatric kidney transplantations are performed, and this is a limitation of all studies conducted in this population [7]. Other limitations include the retrospective nature of the study which made the study prone to selection bias and missing data. As a result, key indicators of intraoperative management including CVP and BP were only available for a subset of 23–25 patients among whom it was tightly controlled. In addition, data on diuretic use intraoperatively and in the immediate post-transplant period was unable to be obtained and its contribution to urine output and early GFR post-transplant was unable to be assessed.

The clinical applicability of the key findings in this study is limited due to the small sample size and retrospective, observational nature. Future studies should investigate alternative targets for perioperative haemodynamic goals. Previous studies have highlighted that CVP and BP correlate poorly with blood volume, and thus, do not necessarily predict organ perfusion and fluid status [33, 34]. Therefore, other measures of cardiac output such as transpulmonary thermodilution may provide a more accurate indication of haemodynamic stability [34] and ensure more appropriate administration of fluids and vasoactive agents in the perioperative period.

The effect of the perioperative period on early graft function in living donor paediatric kidney transplantation was investigated in this study. The results demonstrated that intraoperative and postoperative characteristics, such as CVP, BP, fluid administration and vasoactive agent use, did not affect eGFR in the first month post-transplant. As there was minimal variation in CVP and BP and no negative associations between perioperative characteristics and early graft function were demonstrated, this study suggests that current practices to maintain haemodynamic goals are likely achieving appropriate outcomes. However, better individualisation of intravenous fluid administration may be appropriate in the postoperative period. In conclusion, this exploratory study provides the rationale for future randomised controlled trials to examine the effect of intraoperative and postoperative interventions on graft function in living donor paediatric kidney transplantation. This will inform evidence-based guidelines and protocols to optimise transplant outcomes.

## Supplementary Information

Below is the link to the electronic supplementary material.

Graphical abstract (PPTX 81.1 KB)

Supplementary file1 (DOCX 838 KB)

## Data Availability

The datasets generated during and/or analysed during the current study are available from the corresponding author on reasonable request.
