# Supplementary material for: Effect of perioperative management on early graft function in living donor paediatric kidney transplantation
Source: Pediatr Nephrol. 2024 Sep 16;40(1):231–42. doi: 10.1007/s00467-024-06520-4 (PMC11584495; doi:10.1007/s00467-024-06520-4)
Supplement: Supplementary file 1 — Graphical abstract (PPTX 81.1 KB) [file 467_2024_6520_MOESM1_ESM.pptx]

## Slide 1
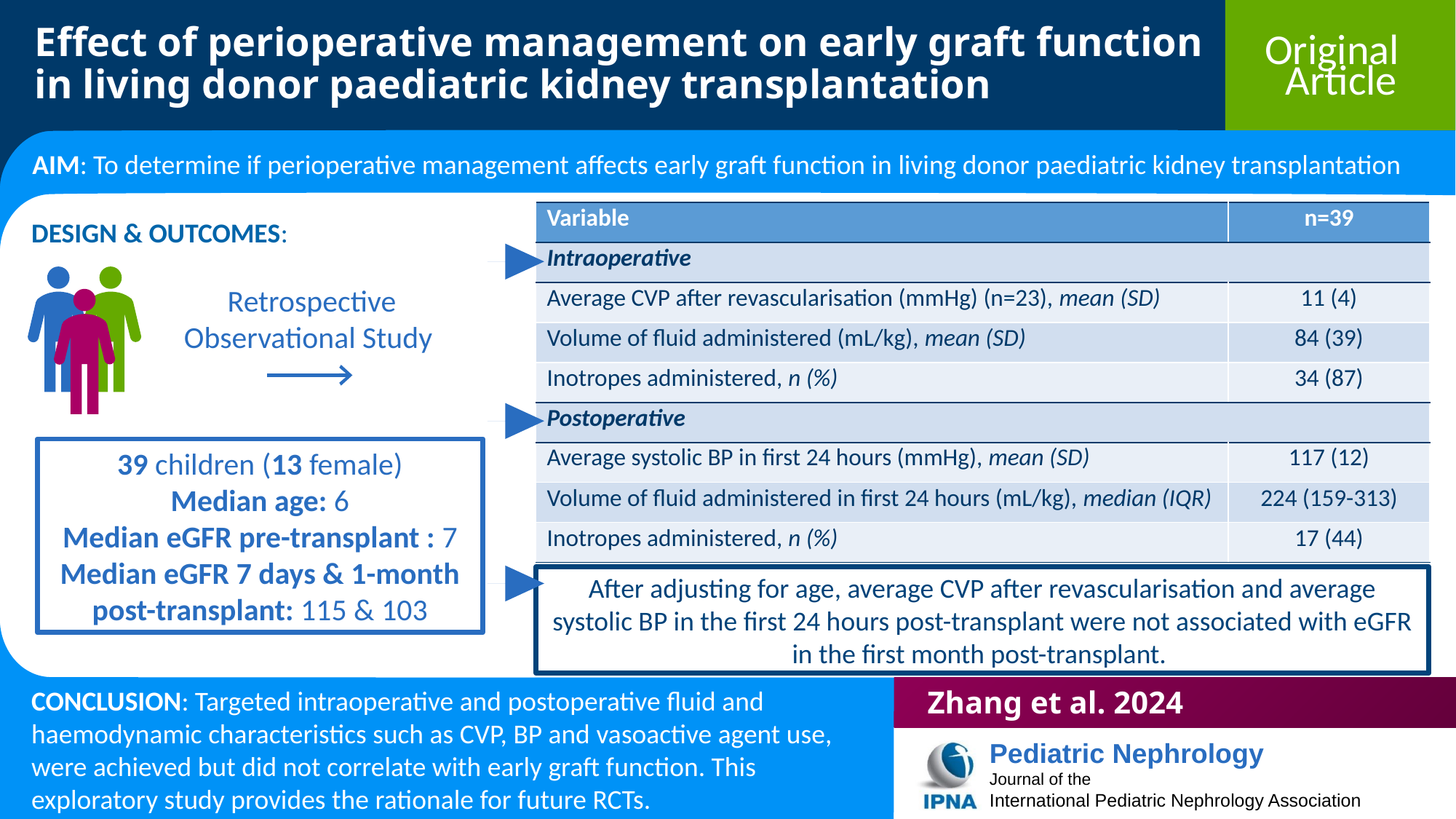

Effect of perioperative management on early graft function in living donor paediatric kidney transplantation
AIM: To determine if perioperative management affects early graft function in living donor paediatric kidney transplantation
| Variable | n=39 |
| --- | --- |
| Intraoperative | |
| Average CVP after revascularisation (mmHg) (n=23), mean (SD) | 11 (4) |
| Volume of fluid administered (mL/kg), mean (SD) | 84 (39) |
| Inotropes administered, n (%) | 34 (87) |
| Postoperative | |
| Average systolic BP in first 24 hours (mmHg), mean (SD) | 117 (12) |
| Volume of fluid administered in first 24 hours (mL/kg), median (IQR) | 224 (159-313) |
| Inotropes administered, n (%) | 17 (44) |
DESIGN & OUTCOMES:
Retrospective Observational Study
39 children (13 female)
Median age: 6
Median eGFR pre-transplant : 7
Median eGFR 7 days & 1-month post-transplant: 115 & 103
After adjusting for age, average CVP after revascularisation and average systolic BP in the first 24 hours post-transplant were not associated with eGFR in the first month post-transplant.
CONCLUSION: Targeted intraoperative and postoperative fluid and haemodynamic characteristics such as CVP, BP and vasoactive agent use, were achieved but did not correlate with early graft function. This exploratory study provides the rationale for future RCTs.
Zhang et al. 2024
