# Supplementary material for: Effect of perioperative management on early graft function in living donor paediatric kidney transplantation
Source: Pediatr Nephrol. 2024 Sep 16;40(1):231–42. doi: 10.1007/s00467-024-06520-4 (PMC11584495; doi:10.1007/s00467-024-06520-4)
Supplement: Supplementary file 2 — Supplementary file1 (DOCX 838 KB) [file 467_2024_6520_MOESM2_ESM.docx]

**Supplementary Information**

**
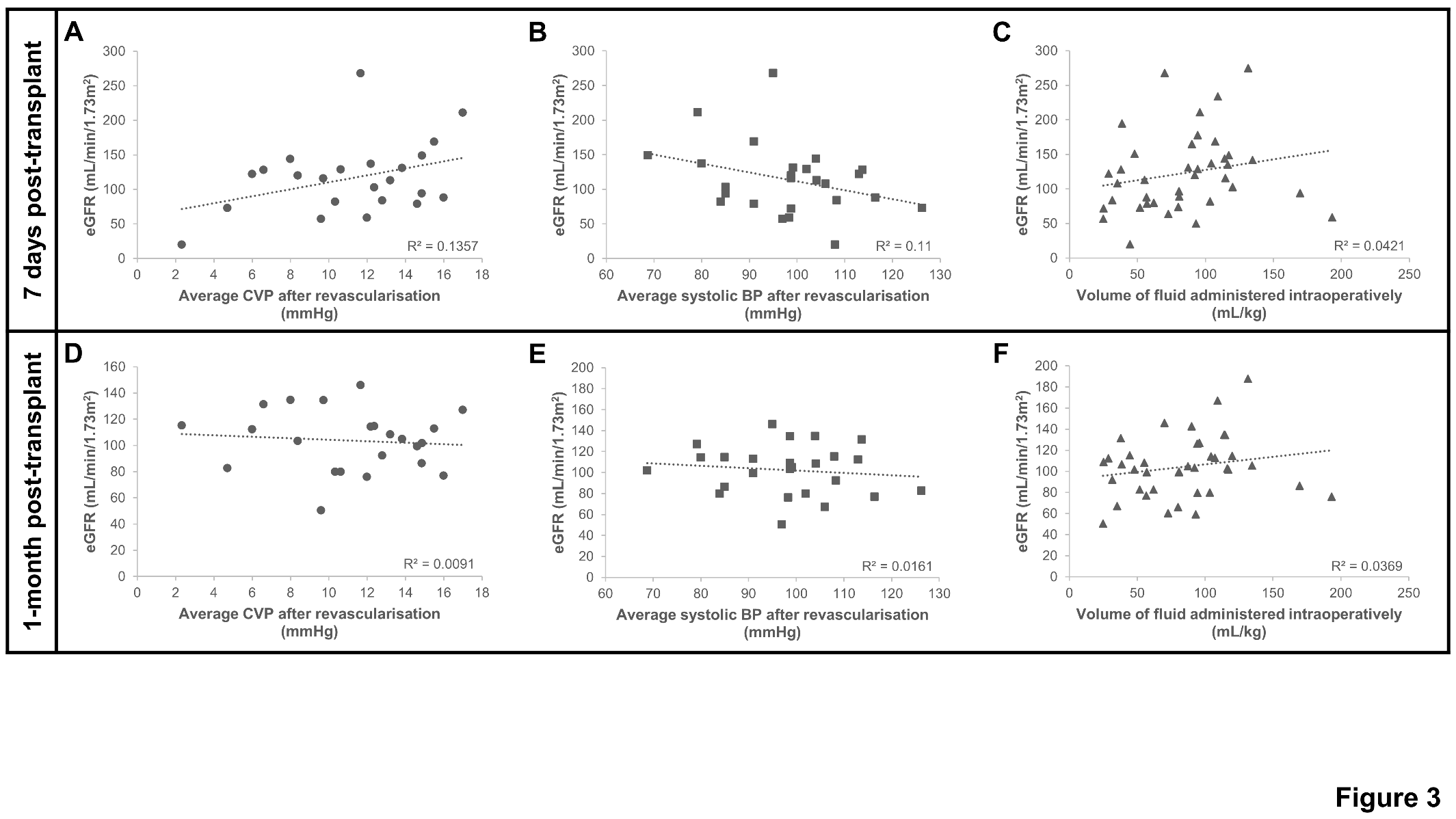
**

**Figure S1.** *Scatter plots demonstrating the relationship between intraoperative characteristics and estimated glomerular filtration rate (eGFR) 7 days and 1-month post-living donor paediatric kidney transplant (n=39).* Correlation between **(A)** average central venous pressure (CVP) after revascularisation (n=23), **(C)** average systolic blood pressure (BP) after revascularisation (n=25), **(C)** volume of fluid administered intraoperatively, and eGFR 7 days post-transplant. Correlation between **(D)** average CVP after revascularisation (n=23), **(E)** average systolic BP after revascularisation (n=25), **(F)** volume of fluid administered intraoperatively, and eGFR 1-month post-transplant.

**
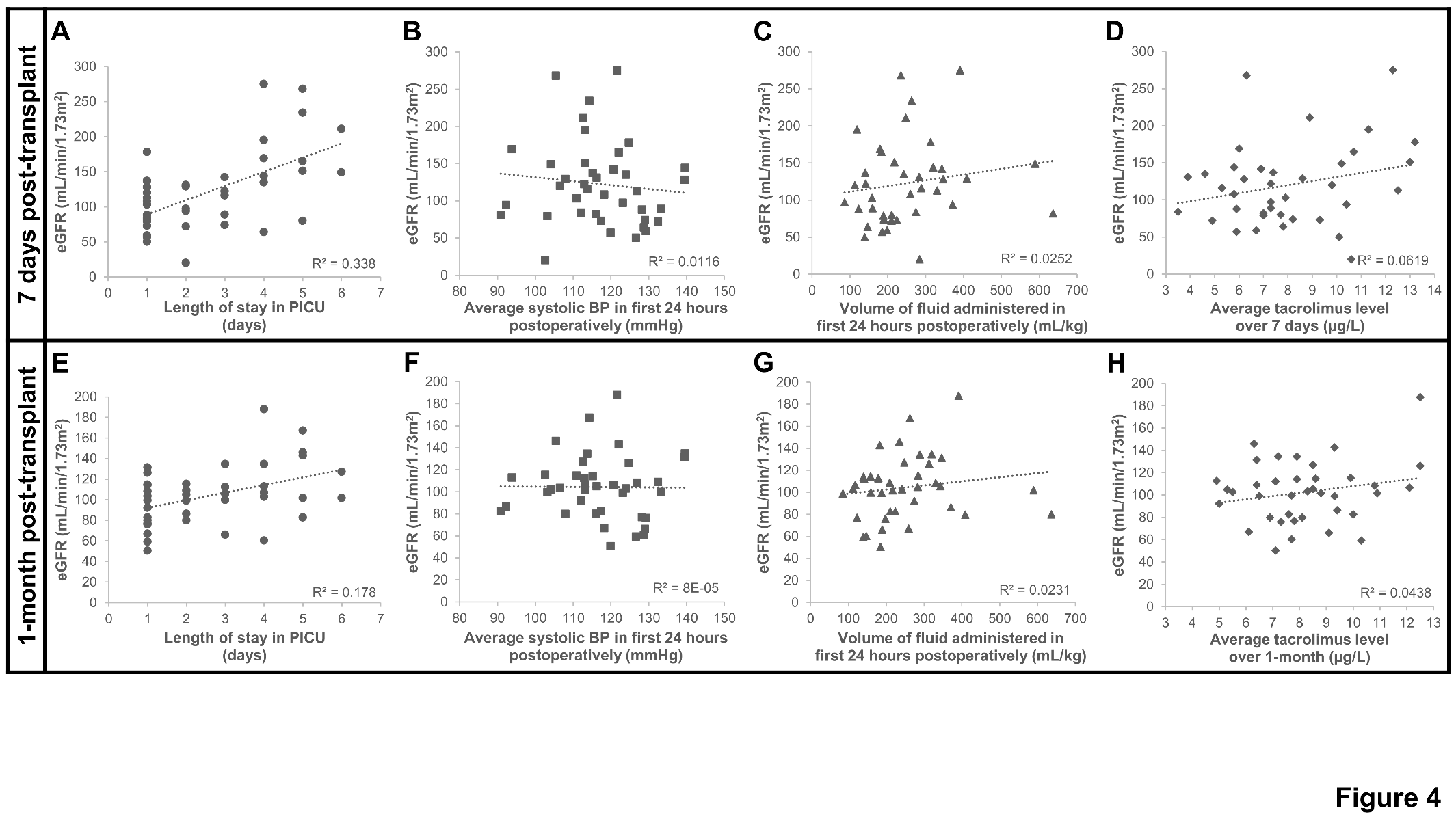
**

**Figure S2.** *Scatter plots demonstrating the relationship between postoperative characteristics and estimated glomerular filtration rate (eGFR) 7 days and 1-month post-living donor paediatric kidney transplant (n=39).* Correlation between **(A)** length of stay in paediatric intensive care unit (PICU), **(B)** average systolic blood pressure (BP) in first 24 hours postoperatively, **(C)** volume of fluid administered in first 24 hours postoperatively, **(D)** average tacrolimus level over 7 days (n=38), and eGFR 7 days post-transplant. Correlation between **(E)** length of stay in PICU, **(F)** average systolic BP in first 24 hours postoperatively, **(G)** volume of fluid administered in first 24 hours postoperatively, **(H)** average tacrolimus level over 1-month (n=38), and eGFR 1-month post-transplant.
